# A novel protein encoded by circHNRNPU promotes multiple myeloma progression by regulating the bone marrow microenvironment and alternative splicing

**DOI:** 10.1186/s13046-022-02276-7

**Published:** 2022-03-08

**Authors:** Xiaozhu Tang, Zhendong Deng, Pinggang Ding, Wanting Qiang, Yue Lu, Shengyao Gao, Ye Hu, Ye Yang, Juan Du, Chunyan Gu

**Affiliations:** 1 Nanjing Hospital of Chinese Medicine affiliated to Nanjing University of Chinese Medicine, Nanjing, China; 2 School of Medicine & Holistic Integrative Medicine, Nanjing University of Chinese Medicine, Nanjing, China; 3 Department of Hematology, Myeloma & Lymphoma Center, Changzheng Hospital, Naval Medical University, Shanghai, China; 4 Department of Radiotherapy, The Affiliated Cancer Hospital of Nanjing Medical University, Nanjing, China

**Keywords:** Multiple myeloma, circHNRNPU, Proliferation, RIP-seq, Marker

## Abstract

**Backgroud:**

Multiple myeloma (MM) is an incurable plasma cell malignancy in the bone marrow (BM), while immunoglobulin D type of MM (IgD MM) is a very rare but most severe subtype in all MM cases. Therefore, systemic study on IgD MM is purposeful to disclose the recurrent and refractory features in both IgD and other types of MM, and beneficial to the development of potent therapeutic strategy on MM.

**Methods:**

Agilent SBC-ceRNA microarray chips were employed to examine 3 normal plasma cell samples (NPCs), 5 lgD MM samples and 5 lgG MM samples, respectively. Sanger sequencing, RNase R digestion and qPCR assays were used to detect the existence and expression of circHNRNPU. BaseScope™ RNA ISH assay was performed to test circHNRNPU levels in paraffin-embedded MM tissues. The protein encoded by circHNRNPU was identified by LC-MS/MS, which was named as circHNRNPU_603aa. The function of circHNRNPU_603aa on cellular proliferation and cell cycle was assessed by MTT test, colony formation assay, flow cytometry and MM xenograft mouse model in vivo. RIP-seq, RIP-PCR and WB analysis for ubiquitination were performed to explore the potential mechanism of circHNRNPU_603aa in MM. Exosomes were isolated from the culture supernatant of MM cells by ultracentrifugation and characterized by Transmission Electron Microscope and WB confirmation of exosomes markers Alix and CD9.

**Results:**

CircHNRNPU was one of the top most abundant and differentially expressed circRNA in IgD MM relative to lgG and NPCs samples. Increased circHNRNPU was associated with poor outcomes in four independent MM patient cohorts. Intriguingly, MM cells secreted circHNRNPU, which encoded a protein named as circHNRNPU_603aa. Overexpressed circHNRNPU_603aa promoted MM cell proliferation in vitro and in vivo, in contrast knockdown of circHNRNPU_603aa by siRNA abrogated these effects. Due to circHNRNPU_603aa including RNA-binding RGG-box region, it regulated SKP2 exon skipping, thereby competitively inhibited c-Myc ubiquitin so as to stabilize c-Myc in MM. MM cells secreted circHNRNPU through exosomes to interfere with various cells in the BM microenvironment.

**Conclusion:**

Our findings demonstrate that circHNRNPU_603aa is a promising diagnostic and therapeutic marker in both MM cells and BM niche.

**Supplementary Information:**

The online version contains supplementary material available at 10.1186/s13046-022-02276-7.

## Background

Multiple myeloma (MM) is a molecularly and cytogenetically heterogeneous hematological malignancy heavily dependent on bone marrow (BM) microenvironment, which is characterized by the clonal proliferation of malignant plasma cells [1]. Despite the advanced development of targeted drugs, such as immune modulators and proteasome inhibitors, have greatly improved outcomes of MM patients over the decades, MM remains incurable [2, 3]. As a most severe type among all the subtypes of MM, immunoglobulin D multiple myeloma (IgD MM) is very rare comprising only 1 to 2% of all MM cases featured by diagnosis in relatively young patients, and often accompanied by multiple adverse prognosis, such as extraosseous lesions, renal failure, extramedullary involvement, amyloidosis [4, 5]. The most distinguish characteristic of lgD MM is poor outcome of refractory status, only 13 ~ 21-month overall survival (OS) compared with 3 ~ 6-year overall median survival in common MM [6–8]. Therefore, systemic investigation on IgD MM is purposeful to reveal the recurrent and refractory features in both IgD and other types of MM, and beneficial to develop potent therapeutic strategy on MM.

The advanced research achievement of the microenvironmental interactions between MM cells and the BM niche, and their roles in the progression of disease and acquisition of drug resistance, has promoted the development of novel therapeutic drugs for MM treatment [9–12]. The intercellular interaction in BM niche through exosomes and circular RNAs (circRNAs) attracts extensive attention [13]. CircRNAs, back-spliced products of exonic or intronic sequence of precursor mRNA (pre-mRNA), are a fascinating class of conserved single-stranded RNA molecules [14–16]. It has been reported that circRNAs act as essential players in cancer initiation, progression and drug resistance [17, 18]. In particular, circRNAs may influence tumor microenvironment through intercellular communication due to its abundance in exosomes and human body fluids [19]. Therefore, circRNAs are now being considered as promising biomarkers for cancer [20]. Many studies have suggested that circRNAs are translatable, which are translated into previously unknown protein isoforms [21–23]. However, the evidences remain insufficient [24], especially in MM.

The research progress of circRNAs inspires us to explore if the malignance within lgD MM counterpart cells is partially driven by circRNAs shuttle in this study. We first discovered the abundant existence of circHNRNPU in IgD MM patients and identified circHNRNPU encoding a novel 603-amino acid protein in MM cells, named as circHNRNPU_603aa. Interestingly, circHNRNPU could be secreted into the BM microenvironment, which was investigated to elucidate its roles in MM in vitro and in vivo. Finally, we revealed a novel downstream target of circHNRNPU_603aa. These findings provide significant insights into the functional importance of circHNRNPU_603aa serving as a promising prognostic and therapeutic target of MM.

## Methods

### Gene expression profiling

GEP cohorts were analyzed in the GEO database as described previously [25, 26]. The total therapy 2 (TT2, GSE2658), the assessment of proteasome inhibition for extending remission (APEX, GSE9782) and the Dutch-Belgian Cooperative Trial Group for Hematology Oncology Group-65 (HOVON65, GSE19784) trial patient cohorts were included. Agilent SBC-ceRNA microarray chips (SBC Human (4*180 K) ceRNA) of IgD MM, IgG MM patient samples and their paired normal samples collected from the Second Affiliated Hospital Hospital of Second Military Medical University were used for high-throughput circRNA sequencing (GSE174510).

### Antibodies and reagents

The primary antibodies were at the dilution of 1:1000, including HNRNPU (16365–1-AP, ProteinTech Group, China), HA (51064–2-AP, ProteinTech Group, China), c-Myc (s1826, Clontech, Japan), Alix (2171S, Cell Signaling Technology, USA), CD9 (13174S, Cell Signaling Technology, USA), Ubiquitin (10201–2-AP, ProteinTech Group, China), β-actin (4970S, Cell Signaling Technology, USA). The goat anti-rabbit IgG/Alexa fluor 647 (BS-0295-G, Bioss, China) was at the 1:200 dilution. The second antibodies goat anti-rabbit IgG(H + L) HRP (FMS-Rb01, Fcmacs) and goat anti-mouse IgG (H + L) HRP (S0002, Affinity) were in 5000 diluted concentrations. GW4869 was purchased from MedChemExpress (Monmouth Junction, NJ, USA).

### Cell lines and cell culture

Human MM cell lines, ARP1 and CAG were cultured in RPMI-1640 (Biological Industries, Israel). HEK-293 cells were cultured in DMEM (Thermo Fisher Scientific, USA). RPMI-1640 and DMEM were supplemented with 10% fetal bovine serum (Gibco, USA), 100 U/mL penicillin, and 100 μg/mL streptomycin (HyClone, USA). The cells were cultured at 37 °C in 5% CO_2_.

### BaseScope™ RNA ISH assay

CircHNRNPU levels in fresh paraffin-embedded tissues obtained from Affiliated Beijing Chaoyang Hospital of Capital Medical University and Affiliated Hospital of Shandong University of Chinese Medicine were analyzed by using BaseScope™ Reagent Kit v2-RED (Advanced Cell Diagnostics, Newark, CA) according to the manufacturer’s instructions as previously described [27]. First, the fresh paraffin-embedded tissues were baked at 60 °C for 1 h before deparaffinized in xylene (2 × 5 min) and ethanol (2 × 2 min), then dried at 60 °C for 2 min. Hydrogen peroxide was used to treat the tissues for 10 min at RT, then target retrieval was introduced for 15 min at 100 °C followed by incubation with protease IV for 30 min at 40 °C. Subsequently, BaseScope probe (BA-Hs-HNRNPU-circRNA-E13E2-Junc targeting 2634–936 of NM_031844.3) was applied for 2 h at 40 °C in a HybEZ oven before incubation with the reagents AMP1 (30 min at 40 °C), AMP2 (30 min at 40 °C), AMP3 (15 min at 40 °C), AMP4 (30 min at 40 °C), AMP5 (30 min at 40 °C), AMP6 (15 min at 40 °C), AMP7 (30 min at RT) and AMP8 (15 min at RT). Finally, circHNRNPU expression was visualized with Fast Red.

### Plasmids and transfection

A commercially available circular RNA expression vector PLC5-ciR (GS0104, Guangzhou Geneseed Biotech Co, China) was used to generate a circ-HNRNPU-overexpression vector. To induce circularization in vivo, side flanking repeat sequences and SA/SD sequences were added to both sides of the 1733 nt sequences (OV-circHNRNPU). The front circular frame contained the endogenous flanking genomic sequences with EcoRI restriction enzyme site, and the back-circular frame included part of the inverted upstream sequence with BamHI site.

Lentiviruses were produced by co-transfection of the expression vector of interest with the packaging plasmids psPAX2 and pMD2G (Addgene) into HEK-293 cells using Hieff Trans™ Liposomal Transfection Reagent (Cat#40802, Yeasen, China). Virus supernatant was collected after 48 h. Transfected MM cells were screened by puromycin. The qPCR and WB methods were performed to examine overexpression efficiency.

### Cell proliferation, colony formation, and cell cycle assays

Cell viability was detected by Thiazolyl Blue Tetrazolium Bromide (MTT) assay (Beijing Solarbio Science & Technology).

For colony formation assay, clonogenic growth was determined by plating 1 × 10^4^ cells in 0.5 mL of 0.33% agar/RPMI 1640 supplemented with 10% FBS. Medium was supplied twice weekly, and cells were cultured for around 2 weeks. Clusters of cells were considered to be a clonogenic colony if over 40 cells. Then the colonies were imaged, and the numbers were counted by using ImageJ.

For cell cycle assay, MM cells were fixed by 70% ethanol, washed with PBS and treated with propidium iodide (PI) solution (Yeasen, China) for 30 min. The samples were analyzed by flow cytometry (Merck Millipore, Germany).

### WB and co-immunoprecipitation (co-IP)

WB was performed as previously described [28]. Co-IP was conducted by using a Pierce Direct Magnetic IP/Co-IP kit (Thermo Scientific) according to the manufacturer’s instructions.

### Immunofluorescent staining and confocal microscopy

Cells were fixed by 4% paraformaldehyde, permeabilized with PBS containing 0.1% Triton X-100, quenched with 50 mM NH_4_Cl (5 min), and blocked with 1% BSA. After overnight incubation with primary antibodies at 4 °C, the slides were incubated with corresponding secondary antibodies. Images were captured by a confocal microscope (TCS SP8, Leica, Germany).

### RNase R treatment

RNase R (Epicentre Biotechnologies, Madison, WI) was used to degrade linear mRNA. In brief, RNAs were extracted from ARP1 and CAG cells, and divided into two parts, one for RNase R digestion and another for control with digestion buffer only. RNase R treatment (20 U/μL) was performed on total RNA (20 μg) at 37 °C for 15 min.

### RT-qPCR

Total RNA was isolated from MM cells using TRIeasyTM Total RNA Extraction Reagent (YEASEN, Shanghai). The Hifair 1st Strand cDNA Synthesis SuperMix was utilized to reverse transcribe 500 ng of purified RNA. PCR samples were prepared with diluted cDNA (1:30), 5 μL SYBR Green PCR master mix (YEASEN, Shanghai) and 0.2 μM each of the forward and reverse primers in a total volume of 10 μL. Quantitative PCR (qPCR) reaction procedure was conducted as follows: predenaturation temperature, 95 °C, 3 min; denaturation temperature, 95 °C, 10 s; annealing temperature, 60 °C, 59 s; a total of 40 cycles. The qPCR was performed in an Analytikjena qPCR soft 4.0 (Gemany). The relative expression level of target genes was calculated by using the 2 − ΔΔCT method and graphed as fold change (2 − ΔΔCT) from control.

### Exosome isolation and identification

Exosomes were collected from the supernatant of MM cells. The supernatant was centrifuged at 300 x g for 10 min, 2000 x g for 10 min, 10,000 x g for 30 min to remove floating cells and debris. The remaining supernatant was centrifuged in an ultracentrifuge at 100,000 x g for 70 min. Then, the collected precipitate was washed with PBS, centrifuged at 100,000 x g for 70 min, and resuspended in 200 μL PBS. Finally, the morphology was characterized by Transmission Electron Microscope (TEM) (Philips TECNAI 20, Netherland). The expressions of Alix and CD9 were detected by WB.

### Mass spectrometry analysis

SDS-PAGE was used to separate proteins, and gel bands at the expected size were excised and digested. The resulting peptides were analyzed by a QExactive mass spectrometer (Thermo Fisher Scientific). Fragment spectra were analyzed according to the NCBI nonredundant protein database.

### RNA immunoprecipitation sequencing (RIP-seq)

RIP experiment was performed as previously described [29]. 5 ~ 20 × 10^6^ cells were collected for protein extraction. Protein A/G MagBeads were pre-coated with 5 μg HA antibody and incubated with cell lysate supernatant. The beads containing immunoprecipitated RNA-protein complex were treated with 150 μL of Proteinase K buffer. Specific binding RNAs were isolated and analyzed by RT-qPCR or high throughput sequencing. RIP-seq assay was performed by Illumina sequencing platform of Novogene Biotechnology Co., Ltd. (Beijing, China) (GSE174501).

### MM-xenografted SCID/NOD mouse model

1 × 10^6^ wild type (WT) and circHNRNPU-overexpression (circHNRNPU-OE) MM cells were injected subcutaneously into the left and right abdominal flanks of 6 ~ 8-week old SCID/NOD mice, respectively.

Tumor diameter was measured daily using the calipers. When the tumor diameter was up to 20 mm, the mice were sacrificed. The tumor tissues were collected, weighed, and photographed. All animal studies were conducted in accordance with the Government-published recommendations for the Care and Use of Laboratory Animals, and approved by the Institutional Ethics Review Boards of Nanjing University of Chinese Medicine (Ethics Registration no. 201905A003).

### Statistical analyses

Statistical analyses were performed using SPSS version 22.0 or GraphPad Prism 6.01 software, and all values were expressed as mean ± SD unless otherwise specified. A two-tailed Student’s t-test (2 groups) or one-way analysis of variance (ANOVA) (≥3 groups) was utilized to evaluate statistical significance. A Kaplan–Meier curve and Log-rank test were employed to determine MM patient survival. *p* < 0.05 was considered statistically significant.

## Results

### CircHNRNPU is the most abundantly and differentially expressed circRNA in IgD MM

**Fig. 1:**
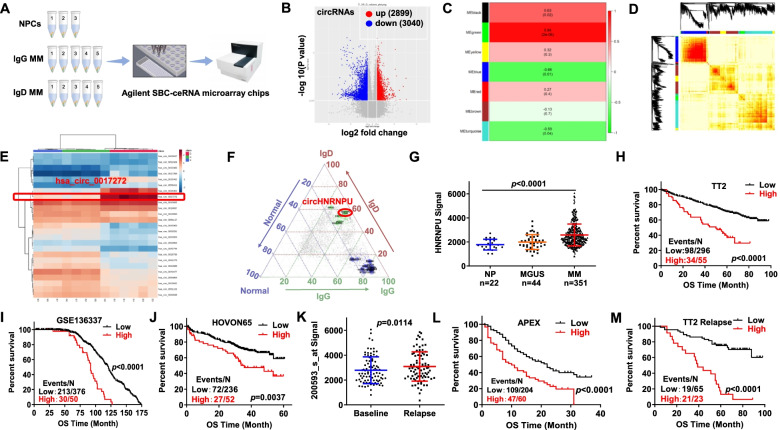
CircHNRNPU is the most abundantly and differentially expressed circRNA in IgD MM. **A** Agilent SBC-ceRNA microarray chips was employed to detect circRNA expression in 3 normal plasma cell samples (NPCs), 5 lgD MM samples and 5 lgG MM samples respectively. **B** The volcano plot of differentially expressed circRNAs between IgD and IgG MM tissues (*n* = 5). X axis, −log10 *P* value; Y aixs, log2 fold change. **C** Seven modules were enriched in the differentially expressed circRNA and mRNA in IgD MM using Combined weighted gene correlation network analysis (WGCNA) (FPKM> 1 and at least expressed in 3 samples). **D** Turquoise and green modules were the genes that highly expressed in IgD MM patients compared with IgG MM samples and their paired normal tissues. **E**-**F** CircHNRNPU (hsa_circ_0017272) was the most abundantly and differentially expressed circRNA in IgD MM compared with IgG MM and their paired normal tissues. **G** HNRNPU mRNA levels were significantly increased in MM samples. The signal level of HNRNPU was shown on the y-axis. Patients were designated as healthy donors with normal bone marrow plasma cells (NP, *n* = 22), monoclonal gammopathy of undetermined significance (MGUS, *n* = 44), or multiple myeloma (MM, *n* = 351), sorted on the x-axis. **H**-**J** Increased HNRNPU mRNA expression was associated with poor overall survival (OS) in MM patients from (H) TT2, (I) GSE136337 and (J) HOVON65 patient cohorts. **K** In paired MM samples collected at first diagnosis and relapse, HNRNPU mRNA expression was increased in the relapsed samples relative to the first diagnosis samples. **L**-**M** Elevated HNRNPU expression was correlated with decreased OS in relapsed patients in (L) APEX and (M) TT2 cohorts

To investigate the most abundantly and differentially expressed circRNA in IgD MM compared to lgG and NPCs samples, we employed Agilent SBC-ceRNA microarray chips to detect circRNA expression in 3 normal plasma cell samples (NPCs), 5 lgD MM samples and 5 lgG MM samples, respectively (Fig. 1A). Intriguingly, a total of 28,456 circRNAs were identified and 5939 were differentially expressed between IgD MM and IgG MM samples (*p* < 0.05 and fold change > 2; 2899 upregulated and 3040 downregulated circRNAs in IgD MM; Fig. 1B; Supplementary differentially expressed genes). Next, we adopted Co-expression Analysis Identifies Gene Networks using combined weighted gene correlation network analysis (WGCNA) to analyze the differentially expressed circRNAs in IgD MM (FPKM > 1, at least expressed in 3 samples). As shown in Fig. 1C, seven modules were enriched, in which the turquoise and green modules were the genes highly expressed in IgD MM patients compared to IgG MM samples and NPCs (Fig. 1D). The turquoise and green modules include 1267 circRNA probes for the differentially expressed genes. Subsequently, hub genes were analyzed by the relief algorithm to screen out 25 qualified probes, and then cluster for unsupervised algorithm. These 25 narrow-down candidates distinguished from the specified groups, and circHNRNPU (hsa_circ_0017272) was in them and highly expressed in IgD MM patients compared to IgG MM and NPCs (Fig. 1E-F).

As circRNA is co-expressed with the corresponding linear mRNA [24], we examined HNRNPU expression in MM GEP cohorts. HNRNPU mRNA was significantly increased in MM cells compared with NP cells and monoclonal gammopathy of undetermined significance (MGUS) cells (Fig. 1G). In addition, high HNRNPU expression was associated with poor outcomes in TT2 (Fig. 1H), GSE136337 (Fig. 1I) and HOVON65 (Fig. 1J) patient cohorts, which included over 1200 MM patients.

IgD MM has a poorer prognosis relative to other MM isotypes [30], we assumed that HNRNPU might be a biomarker for high-risk MM. HNRNPU expression was increased in MM relapse samples relative to first-diagnosis MM samples in 88 paired patient samples (Fig. 1K). HNRNPU expression was also elevated in relapse MM samples from APEX (Fig. 1L) and TT2 cohorts (Fig. 1M) compared with newly diagnostic counterparts (*p* < 0.05). The detailed information of patient cohorts was included in Supplementary information of patient cohorts.

### Validation of the existence and expression of circHNRNPU in IgD MM

**Fig. 2:**
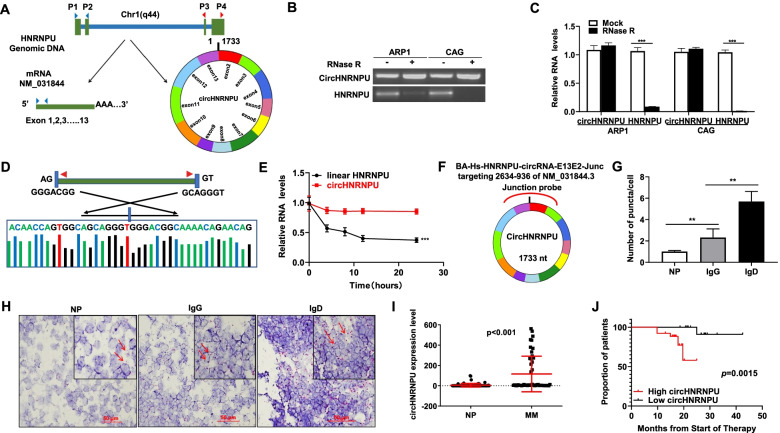
Validation of the existence and expression of circHNRNPU in IgD MM. **A** Illustration of the annotated genomic region of HNRNPU, the putative different RNA splicing forms and the validation strategy for circular exon 2 to 13 (circHNRNPU). Convergent (blue) and divergent (red) primers were designed to amplify the linear or back-splicing products. **B**-**C** RNA levels of circHNRNPU and linear HNRNPU ± RNase R were determined by RT-PCR and qRT-PCR. **D** Sanger sequencing following PCR was conducted using the indicated divergent flanking primers confirmed the “head-to-tail” splicing of circHNRNPU in ARP1 and CAG cells. **E** Relative RNA levels of circHNRNPU and HNRNPU in different time points. **F** BaseScope probe was applied for detecting circHNRNPU expression in healthy controls, IgG and IgD MM patients. **G**-**H** The levels of circHNRNPU in IgD patients were superior than IgG patients and normal control (NP) evaluated by RNA scope analysis, and the representative staining images were shown with positive reactions indicated by red arrows. **I** CircHNRNPU levels were significantly elevated in MM patients. **J** Elevation of circHNRNPU was associated with inferior EFS survival. The data are presented as mean ± SD.*p < 0.05, **p < 0.01, ***p < 0.001

In order to verify that the exons 2 and 13 of HNRNPU gene formed the endogenous circHNRNPU (hsa_circ_0017272), convergent and divergent primers were designed to detect the linear and back-spliced forms of HNRNPU, respectively (Fig. 2A). Upon RNase R digestion, linear HNRNPU was significantly degraded (*p <* 0.001), while circHNRNPU was resistant to degradation, confirming the existence of circHNRNPU (Fig. 2B-C). In addition, Sanger sequencing recognized the circHNRNPU junction site (Fig. 2D), which further proved the presence of circHNRNPU in MM cells. CircHNRNPU showed a longer half-life compared with its linear counterpart (Fig. 2E).

Next, we performed a BaseScope™ RNA ISH assay in IgG and IgD MM patient tissues by using a commercial circRNA junction probe (1ZZ probe named BA-Hs-HNRNPU-circRNA-E13E2-Junc targeting 2634–936 of NM_031844.3) to detect the expression of circHNRNPU (Fig. 2F). The abundance of circHNRNPU in IgD tissues was much higher than that in IgG and healthy control (NP) tissues (Fig. 2G-H). Furthermore, we collected blood samples from 48 MM patients and 48 healthy controls, and extracted the total RNA for testing the expression of circHNRNPU. Intriguingly, circHNRNPU was substantially more abundant in MM patients than healthy controls (*p* < 0.001) (Fig. 2I), and MM patients with higher circHNRNPU expression exhibited a significantly inferior EFS survival (*p <* 0.01) (Fig. 2J), suggesting that circHNRNPU might be a potential biomarker of IgD MM progression.

### CircHNRNPU encodes a novel HNRNPU isoform

**Fig. 3:**
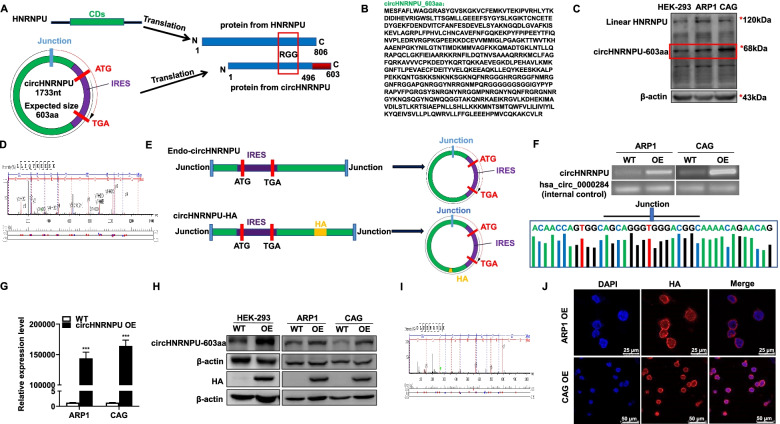
CircHNRNPU encodes a novel HNRNPU isoform. **A** The putative open reading frame (ORF) in circHNRNPU. The sequences of the putative ORF were shown in green, internal ribosomal entrance site (IRES) sequences were shown in purple. **B** The predicted sequence of circHNRNPU_603aa. **C** WB analysis of endogenous circHNRNPU_603aa expression in HEK-293, ARP1 and CAG cells. **D** The specific peptides from circHNRNPU_603aa were identified by mass spectrometry analysis. **E** Illustration of endo-circHNRNPU and circHNRNPU-HA. **F**-**G** RNA levels of circHNRNPU and linear HNRNPU were determined by RT-PCR and qRT-PCR. Sanger sequencing following PCR was conducted using the indicated divergent primers to confirm the precise splicing of circHNRNPU-OE plasmid in ARP1 and CAG cells. The hsa_circ_0000284 was used as internal control gene. **H** WB analysis of circHNRNPU_603aa overexpression in HEK-293, ARP1 and CAG cells detected by HNRNPU and HA tag antibody. **I** The specific peptides from circHNRNPU_603aa were identified by mass spectrometry analysis. **J** Confocal microscope live image was used to capture circHNRNPU_603aa cellular localization. Scale bars, 25 μm. The data are presented as mean ± SD.*p < 0.05, **p < 0.01, ***p < 0.001

Growing evidences have been shown that circRNAs could serve as the templates for protein or peptide [31]. However, it is still not clear whether circRNAs can be translated to novel proteins and possess biological activity in MM. Therefore, we analyzed the putative open reading frame (ORF) of circHNRNPU in circRNADb. We found a putative internal ribosome entry site (IRES) sequence (from + 201 to + 374) of circHNRNPU in the ORF with the potential to encode a 603-aa peptide containing RNA-binding RGG-box region, an RNA binding motif and a predictor of RNA binding activity [32–34]. In human circHNRNPU, the tandem “AUG” within the RNA circle could start the translation of a novel protein. The analysis of ORF indicated that it took more than one whole circle of circHNRNPU to translate the novel 603-aa protein (Fig. 3A), which we termed as “circHNRNPU_603aa” (Fig. 3B). To validate endogenous circHNRNPU could be translated into circHNRNPU_603aa, we exploited a commercial HNRNPU antibody to recognize the N-terminus of HNRNPU as to confirm the existence of circHNRNPU_603aa. Inspiringly, WB method successfully recognized circHNRNPU_603aa at the expected size in HEK-293, ARP1 and CAG cells (Fig. 3C). In addition, the MS analysis also identified the specific peptide fragments from circHNRNPU_603aa, which further validated the coding ability of endogenous circHNRNPU (Fig. 3D).

To testify the function of circHNRNPU_603aa, we inserted the full-length sequence of circHNRNPU into a plasmid linked with HA tag. Since more than one circle of circHNRNPU translation is required to complete the process, we added the HA tag behind the termination codon TGA, which would not affect its translation mode or protein functional domain (Fig. 3E). Next, we transduced the plasmid into MM cells using lentiviruses. Subsequently, qPCR confirmed that circHNRNPU was overexpressed in ARP1 and CAG cells (Fig. 3F-G). Sanger sequencing following with PCR was conducted to determine the accuracy of the cyclization product (Fig. 3F). Then WB analysis confirmed that the expression of circHNRNPU_603aa was elevated in HEK-293, ARP1 and CAG circHNRNPU-OE cells as expected (Fig. 3H). In addition, HA antibody application and MS analysis further proved the specific peptide fragments from circHNRNPU_603aa (Fig. 3H-I). Furthermore, we tested the cellular localization of circHNRNPU_603aa by using HA antibody, the immunofluorescence results showed that circHNRNPU_603aa was located in both the cytoplasm and nucleus (Fig. 3J).

### CircHNRNPU_603aa promotes MM cell proliferation and clonal expansion

**Fig. 4:**
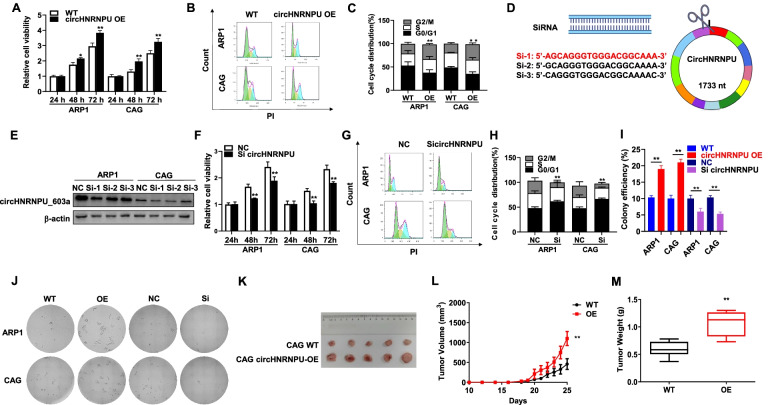
CircHNRNPU_603aa promotes MM cell proliferation and clonal expansion. **A** MTT assay demonstrated higher cell proliferation of circHNRNPU-OE cells compared to WT cells. A two-tailed Student’s t-test was utilized to evaluate statistical significance. **B**-**C** Cell cycle analysis revealed that the proportion of G2/M phase was significantly increased in circHNRNPU-OE cells relative to WT cells. **D** Three siRNAs were designed to target the unique sequence of circHNRNPU, and si-1 was marked in red. **E** WB analysis confirmed the reduction of circHNRNPU_603aa in ARP1 and CAG cells upon transfection with three siRNAs, in which Si-1 had a desired inhibitory effect. **F** Decreased circHNRNPU_603aa resulted in lower cell proliferation rate in ARP1 and CAG cells detected by MTT. A two-tailed Student’s t-test was utilized to determine statistical significance. **G**-**H** Cell cycle analysis revealed that the proportion of G2/M phase significantly decreased in si-circHNRNPU cells relative to NC cells. **I**-**J** Images of representative soft agar plates showed more colonies formed by circHNRNPU-OE cells and less colonies formed by si-circHNRNPU cells compared with control cells. **K** Photographic images of xenograft mice at day 25 and xenografts from NOD-SCID mice. **L** Time course of tumor growth in NOD-SCID mice. **M** Tumor weight in WT and circHNRNPU-OE group at day 25 after injection of MM cells. The data are presented as mean ± SD.*p < 0.05, **p < 0.01, ***p < 0.001

We performed MTT, cell cycle and colony formation assays in circHNRNPU_603aa-OE MM cells to further determine if circHNRNPU_603aa was a contributing factor to MM progression. Cell proliferation rate was increased in circHNRNPU_603aa-OE cells compared with WT cells in both ARP1 and CAG cells, as demonstrated by MTT assay (Fig. 4A). Cell cycle analysis also showed a significantly increased proportion of G2/M phase in circHNRNPU_603aa-OE cells (Fig. 4B-C). In addition, three siRNAs were designed to target the specific peptide fragments in circHNRNPU_603aa (Fig. 4D). CircHNRNPU_603aa expression was decreased upon transfection of three siRNAs respectively, especially siRNA-1 (Si-1) had a desired inhibitory effect that was used in the following study (Fig. 4E). MTT assay demonstrated that the proliferation of ARP1 and CAG cells was significantly suppressed upon silencing circHNRNPU_603aa (*p* < 0.01) (Fig. 4F). Flow cytometry analysis detected that the G2/M phase was significantly decreased upon interfered by Si-1 in MM cells (Fig. 4G-H). Consistently, the colony formation assay showed that circHNRNPU_603aa constructively overexpression or interfered by Si-1significantly altered the long term cell growth of MM cells, which were indicated by increased or reduced colony formation in both ARP1 and CAG cells, respectively (Fig. 4I-J & Supplementary images of colony formation). To further validate the effect of circHNRNPU_603aa on MM proliferation in vivo, CAG WT and circHNRNPU-OE cells were injected subcutaneously into the right or left flanks of NOD-SCID mice, respectively. Tumors formed by circHNRNPU-OE cells grew more rapidly than those formed by WT cells, with significantly increased tumor weight and volume (*p* < 0.05) (Fig. 4K-M). Taken together, these findings indicated that circHNRNPU_603aa promoted MM cell proliferation and clonal expansion.

### Identification of circHNRNPU_603aa-regulated alternative splicing events

**Fig. 5:**
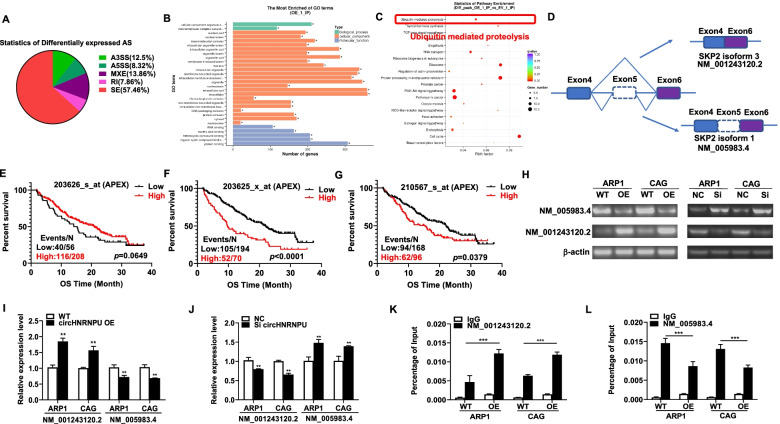
Identification of circHNRNPU_603aa-regulated AS events. **A** Number of AS events in each category. **B** GO Molecular Function enrichment of circHNRNPU_603aa-regulated AS genes. **C** The pathway enrichment analysis of above genes revealed that their molecular function was centered on ubiquitin mediated proteolysis. **D** The expression of SKP2 exon 5 skipping splice variants was significantly increased by increased circHNRNPU_603aa and ranked top of the circHNRNPU_603aa-regulated AS events. **E**-**G** Survival proportions of patients corresponding to different probes of SKP2 in APEX cohort. 203626_s_at was related to better survival compared with other probes. **H**-**J** The expression of SKP2-NM_001243120.2 in circHNRNPU_603aa-OE and si-circHNRNPU_603aa cells relative to control cells. A two-tailed Student’s t-test was utilized to evaluate statistical significance. **K**-**L** Overexpression of circHNRNPU-603aa directly upregulated SKP2-NM_001243120.2 and downregulated SKP2-NM_005983.4 detected by RIP-qPCR. The data are presented as mean ± SD.*p < 0.05, **p < 0.01, ***p < 0.001

As circHNRNPU_603aa contains RNA-binding RGG-box region, RNA binding motif and a predictor of RNA binding activity [32–34], we employed RNA immunoprecipitation sequencing (RIP-seq) using HA antibody as bait to determine a total of 1069 alternative splicing (AS) events with significant differences, among which exon skipping events accounted for 57.46% (678/1069) (Fig. 5A). We subsequently performed Gene Ontology (GO) function significance enrichment analysis for better understanding of the potential biological effects of these circHNRNPU_603aa-regulated AS events, which were mainly related to cell composition, binding and other functions (Fig. 5B). The pathway enrichment analysis of these circHNRNPU_603aa regulated AS events revealed that their molecular function was centered on ubiquitin mediated proteolysis (Fig. 5C). To further identify the regulation mechanism of circHNRNPU_603aa for AS, we performed de novo discovery of the circHNRNPU_603aa binding motif using the overlap exon sequences of circHNRNPU_603aa regulated AS events and circHNRNPU_603aa binding transcripts from RIP-seq data. Among these circHNRNPU_603aa regulated AS events, the expression of SKP2 exon 5 skipping splice variants was significantly increased in circHNRNPU_603aa-OE cells and ranked the top of AS events (Fig. 5D). SKP2 encodes a member of the F-box protein family, which is characterized by F-box nearly 40 amino acid motif. The F-box proteins constitute one of the four subunits of ubiquitin protein ligase complex called SCF complex, which plays an important role in ubiquitination [35–37] and might be related to the molecular function of circHNRNPU_603aa.

In particular, we found that the probe 203626_s_at, designed on the basis of SKP2- NM_005983.4 splicing variant, was associated with better overall survival compared with other probes in APEX cohort (Fig. 5E-G). We detected the expressions of the two isoforms SKP2-NM_005983.4 and SKP2-NM_001243120.2 in circHNRNPU_603aa-OE cells by qPCR. Interestingly, the expression of SKP2-NM_001243120.2 was increased in circHNRNPU_603aa-OE cells relative to WT cells in both ARP1 and CAG cells (Fig. 5H-I). In comparison, the expression of SKP2-NM_001243120.2 was significantly decreased in si-circHNRNPU_603aa cells compared with NC cells treated with non-targeted siRNA (Fig. 5H-J). Furthermore, we adopted RIP-PCR to confirm circHNRNPU-603aa-regulated exon skipping in MM cells using HA antibody as bait. It was found that circHNRNPU-603aa directly bound to the endogenous SKP2-NM_001243120.2, and elevated circHNRNPU-603aa increased SKP2-NM_001243120.2 expression compared with WT cells (Fig. 5K-L). Collectively, we inferred that circHNRNPU-603aa regulated SKP2 exon skipping, thereby spliced NM_005983.4 into NM_001243120.2, suggesting that circHNRNPU-603aa-regulated SKP2 exon skipping might play an important role in promoting MM progression.

### Aberrant splicing of SKP2 contributes to the reduction of c-Myc ubiquitin

**Fig. 6:**
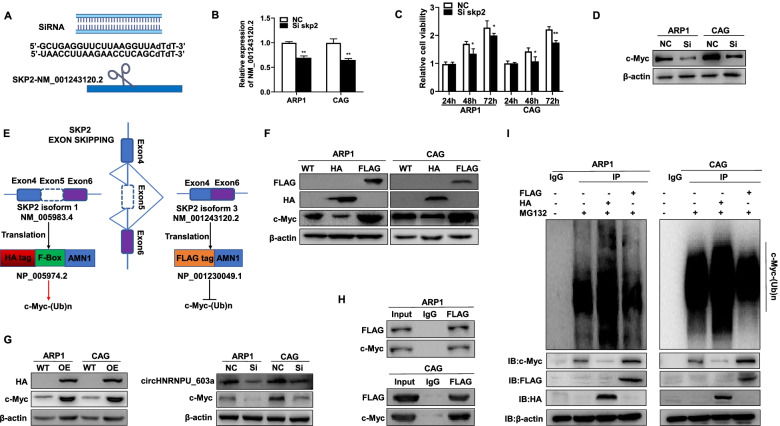
Aberrant splicing of SKP2 contributes to the reduction of c-Myc ubiquitin. **A** Graphic illustration of siRNA targeting SKP2-NM_001243120.2. **B** qPCR analysis of SKP2-NM_001243120.2 expression in ARP1 and CAG cells. **C** Inhibition of SKP2-NM_001243120.2 prominently decreased cell proliferation in ARP1 and CAG cells detected by MTT. **D** WB showed that c-Myc expression was decreased in si-SKP2-NM_001243120.2 cells compared with NC cells. **E** Graphic illustration of SKP2-NM_005983.4-OE and SKP2-NM_001243120.2-OE plasmids linked with HA and FLAG tag, respectively. **F** SKP2-NM_001243120.2 FLAG-tagged isoform upregulated c-Myc expression while the SKP2-NM_005983.4 HA-tagged isoform downregulated c-Myc expression in ARP1 and CAG cells. **G** WB confirmed that c-Myc expression was increased in circHNRNPU_603aa-OE cells and downregulated in si-circHNRNPU_603aa cells. **H** Co-IP experiment indicated an interaction between SKP2 and c-Myc in MM cells. **I** SKP2-NM_001243120.2 FLAG-tagged isoform or SKP2-NM_005983.4 HA-tagged isoform overexpressed cells were treated with MG132 for 12 h. An ubiquitination assay was performed using anti-c-Myc magnetic beads, and the ubiquitylated proteins were detected by Ub antibody. The data are presented as mean ± SD.*p < 0.05, **p < 0.01, ***p < 0.001

To further understand the significance of circHNRNPU-603aa-regulated SKP2 exon skipping and clarify the function of the two splicing isoforms of SKP2 in MM, we first designed a siRNA targeting SKP2-NM_001243120.2 (Fig. 6A-B). As shown in Fig. 6C, the cell proliferation was decreased in si-SKP2-NM_001243120.2 cells relative to NC cells. Since SKP2 is associated with SCF complex participating in c-Myc proteosomal degradation [36], our WB analysis identified that c-Myc expression was decreased in si-SKP2-NM_001243120.2 cells relative to NC cells (Fig. 6D).

As the protein encoded by SKP2-NM_001243120.2 lacks F-Box domain compared with the protein encoded by SKP2-NM_005983.4, which is a component of a SCF (SKP1-CUL1-F-box protein) E3 ubiquitin-protein ligase complex mediating the ubiquitination and subsequent proteasomal degradation of c-Myc [36], we further constructed SKP2-NM_005983.4-OE and SKP2-NM_001243120.2-OE plasmids linked with HA and FLAG tags respectively to examine the molecular function of the two splicing isoforms of SKP2 (Fig. 6E). Intriguingly, SKP2-NM_001243120.2 FLAG-tagged isoform upregulated c-Myc expression, while SKP2-NM_005983.4 HA-tagged isoform downregulated c-Myc expression (Fig. 6F). In addition, c-Myc expression was increased in circHNRNPU_603aa-OE cells and decreased in si-circHNRNPU_603aa cells relative to control cells (Fig. 6G). Co-IP assay validated the interaction between SKP2 and c-Myc in MM cells (Fig. 6H). After MM cells were incubated with 20 μM MG132 (a proteasome inhibitor) for 12 h, the ubiquitination was significantly suppressed in SKP2-NM_001243120.2-OE cells compared with SKP2-NM_005983.4-OE cells (Fig. 6I). Therefore, it was speculated that circHNRNPU-603aa mediated the alternative splicing of SKP2 to upregulate circHNRNPU-603aa splicing isoform SKP2-NM_001243120.2, and subsequently competitively inhibited c-Myc ubiquitination and stabilized c-Myc expression (Fig. 6H-I).

### MM cells secrete circHNRNPU into the BM microenvironment through exosomes

**Fig. 7:**
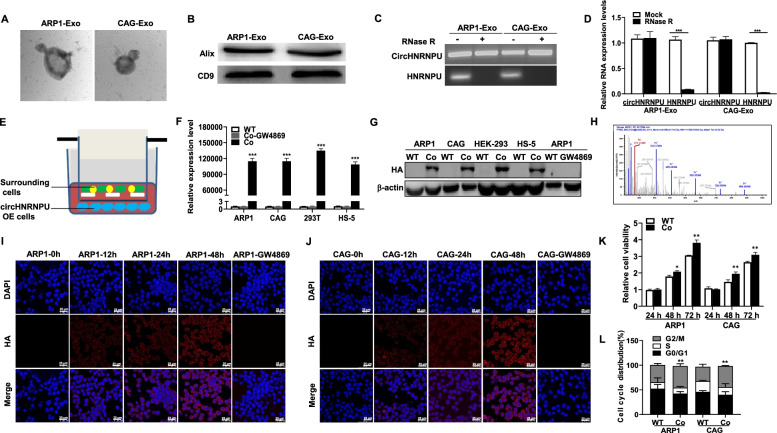
MM cells secrete circHNRNPU into the BM microenvironment through exosomes. **A** Transmission electron microscopy (TEM) was used to characterize exosomes from the culture supernatant of ARP1 and CAG cells. **B** WB analysis showed the presence of exosome markers Alix and CD9. **C**-**D** RNA levels of circHNRNPU and linear HNRNPU ± RNase R were determined by RT-PCR and qRT-PCR. **E** Graphic illustration of cocultured WT ARP1, CAG, HEK-293, HS-5 cells with the CAG circHNRNPU-OE cells using transwell. **F** qPCR analysis of circHNRNPU expression in ARP1, CAG, HEK-293, HS-5 cells cocultured with CAG circHNRNPU-OE cells. **G** WB analysis of circHNRNPU_603aa expression in ARP1, CAG, HEK-293, HS-5 cells cocultured with CAG circHNRNPU-OE cells using HA tag antibody. **H** The specific peptides from circHNRNPU_603aa were identified by mass spectrometry analysis. **I**-**J** Representative confocal images for HA and DAPI showed that circHNRNPU_603aa expression was in time-dependent manner in (I) ARP1 and (J) CAG cells. **K** MTT assay indicated higher cell proliferation rate of cocultured circHNRNPU-OE MM cells than WT cells. **L** Cell cycle assay exhibited higher G2/M proportion of cocultured circHNRNPU-OE MM cells than WT cells. The data are presented as mean ± SD.*p < 0.05, **p < 0.01, ***p < 0.001

**Fig. 8:**
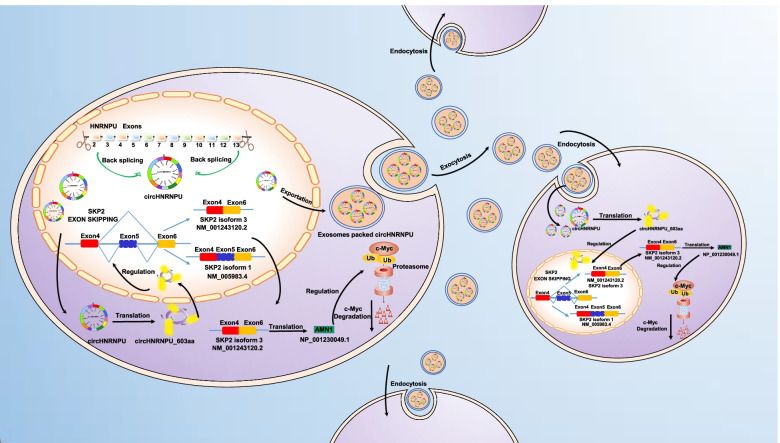
Schematic depiction illustrates that MM cells secrete circHNRNPU into BM microenvironment to regulate SKP2 exon skipping and thereby inhibit c-Myc ubiquitin

It is well known that the BM microenvironment is especially important for the oncogenic growth of MM cells, and many studies have explored the effect of circRNAs on the BM microenvironment through intercellular communication [38]. CircRNAs are abundant and stable in exosomes serving as potential biomarkers for cancer detection and transferring biological activity to recipient cells [39]. We extracted the exosomes from the culture supernatant of ARP1 and CAG cells, which were identified by TEM method (Fig. 7A) and WB confirmation of exosomes markers Alix and CD9 (Fig. 7B). As expected, circHNRNPU was detected in exosomes (Fig. 7C-D). We cocultured WT ARP1, WT CAG, HEK-293 and HS-5 cells with CAG circHNRNPU-OE cells using transwell (Fig. 7E), then we found that all the cocultured cells expressed circHNRNPU (Fig. 7F). Under the treatment with GW4869, a well-recognized exosomes inhibitor that could reduce exosomes release [40], circHNRNPU did not migrate into cells in the BM, indicating that circHNRNPU was secreted by MM cells through exosomes (Fig. 7F). Furthermore, application of HA antibody and MS analysis confirmed the specific peptide fragments from circHNRNPU_603aa in the cocultured cells (Fig. 7G-H). After ARP1 and CAG WT cells were cocultured with CAG circHNRNPU-OE cells for 12 h, 24 h, 48 h, IF staining for HA and DAPI showed that the cocultured cells expressed circHNRNPU_603aa in a time-dependent manner (Fig. 7I-J). As depicted in Fig. 7K, the proliferation rate of cocultured ARP1 and CAG cells was significantly increased (*p* < 0.01) relative to non-cocultured WT cells. Cell cycle analysis also indicated an increased proportion of G2/M phase in cocultured ARP1 and CAG cells relative to non-cocultured cells (Fig. 7L). Summarily, we demonstrated that MM cells could secrete circHNRNPU through exosomes to interfere with various cells in the BM microenvironment (Fig. 8).

## Discussion

MM remains an incurable hematologic malignancy due to adverse features, clonal heterogeneity and BM dependency, in which IgD MM is a very rare but most severe subtype [6]. In all MM cases, the low prevalence and insensitivity to diagnostic methods of IgD MM make it intractable [7]. Therefore, effective therapeutic strategies to target both MM cells and BM niche are of great importance to disclose the recurrent and refractory features in IgD and other types of MM. Diagnosis of IgD MM is difficult, since IgD presents minimal or even undetectable M-protein spikes via serum protein electrophoresis (SPEP) [41, 42]. Thus, some cases manifest as hypogammaglobulinemia or present normal SPEP results, which may lead to misdiagnosis of patients in this subgroup [43, 44]. Many studies have focused on IgD MM and circRNAs, which are novel RNA molecules with significant biological functions, therapeutic and diagnostic significance, especially on cellular interaction in the BM niche [45, 46].

In this study, we employed Agilent SBC-ceRNA microarray chips to identify circHNRNPU as the most abundantly and differentially expressed circRNA in IgD MM samples relative to IgG MM samples and NPCs. BaseScopeTM RNA ISH follows a similar workflow and principle to the well-established novel RNAscopeTM RNA ISH technique used for detection of RNA in situ, with hybridization and amplification of target RNA [47, 48]. It needs an extra step of amplification and a single Z pair target probe instead of the 20 ZZ pair probes employed in the RNAscopeTM technique [49]. We executed BaseScopeTM RNA ISH assay in a tissue microarray with IgG and IgD MM samples using a 1ZZ circRNA junction probe to detect the expression of circHNRNPU. The results showed that the abundance of circHNRNPU in IgD MM was much higher than that in IgG MM and NPCs. Inspiringly, circHNRNPU could be detected in a very small volume of 100 μL blood samples from 48 MM patients. CircHNRNPU was extremely more abundant in IgD MM samples, and the MM patients with higher circHNRNPU expression had a significantly inferior EFS survival. CircRNAs are widely distributed in plasma, urine, tissue samples, cell-free saliva and other human components in a cell-specific manner [50, 51]. The characteristics of circRNAs, such as high and selective abundance, high stability, high conservation and specific expression, may partly explain the potential of circRNAs acting as diagnostic biomarkers [52]. It is conceivable that circHNRNPU may be a potential diagnostic biomarker for IgD MM early detection, precise treatment and prognosis prediction. The contribution of clonal heterogeneity to IgD and other types of MM progression and drug resistance is increasingly being recognized [4]. Comprehensive high-resolution genomic studies have shed new light on the clonal composition of MM at diagnosis and during disease progression. In contrast with what has been postulated one or two decades ago, MM is not derived from one single tumor stem cell, but composed of clonally diverse subsets of MM cells harboring an immense genetic diversity. Different MM clones evolve during the natural course of disease and the shifts in dominant and subdominant clones during therapy and relapse are a fascinating area of MM research [53, 54]. Changes in clonal dynamics over time during MM progression and drug therapy lead to drug resistance and relapse [55]. Our work has explained that MM cells secrete circHNRNPU into BM microenvironment through exosomes to influence the surrounding cells, and the exact composition and distribution of circRNAs contribute to the clonal evolution. Here, we infer that circRNAs shuttle may play a vital role in clonal competition and therefore lead to treatment failure and relapse in IgD and other types of MM.

The common function model of circRNAs is serving as a miRNA sponge and interacts with associated proteins [56]. Recently, it is reported that circZNF609 and circMbl are translatable [57, 58]. Unlike mRNAs, circRNAs can be translated via N6-methyladenosine modification or through internal ribosome entry site (IRES) to promote direct binding of initial factors to circRNAs [59–62]. Intriguingly, many circRNAs that can be translated into proteins have been confirmed to participate in tumor pathophysiology. For example, Yang et al. [56] found the existence of FBXW7-185aa, a 21 KDa protein, which repressed glioma tumorigenesis. In present study, we first characterized that circHNRNPU was translatable and encoded a novel isoform circHNRNPU_603aa. CircHNRNPU_603aa is translated from the spanning junction ORF formed by the covalent connection of exon 2 and exon 13 of the HNRNPU gene. Particularly, circHNRNPU_603aa has distinct amino acid sequence and contains the RGG region, which is responsible for alternative splicing, compared with the linear mRNA translated protein.

Emerging evidences have shown that alternative splicing is a critical component of post-transcriptional process involving most of the eukaryotic genes that contribute to cell differentiation, and it is closely related to the occurrence and development of tumors and largely responsible for the proteome diversity during tumor development owing to the alternative mRNA transcripts encode structurally or functionally disparate protein [63–65]. In cancer cells, normal AS regulation is disrupted resulting in cancer-specific RNA transcription profiles that further promote cell proliferation, drug resistance and metastasis [66]. The members of HNRNP protein family, such as HNRNPA1 and HNRNPA2, are important regulators of AS. We performed RIP-seq to identify 1187 circHNRNPU_603aa-regulated AS events and found that the molecular function centered on the ubiquitin mediated proteolysis pathway. Following-up exploration revealed that circHNRNPU_603aa regulated SKP2 exon skipping to inhibit c-Myc ubiquitin and stabilize c-Myc expression. The ubiquitin-proteasome system (UPS) regulates the levels and activities of a multitude of proteins working on cell cycle, gene expression, cell survival, cell proliferation and apoptosis in MM [67]. Patients with c-Myc translocation have worse progression-free survival (PFS) and overall survival (OS) [68]. In addition, c-Myc alteration is proposed to be a trigger of monoclonal gammopathy of undetermined significance (MGUS) to MM transition [5], and it is regarded as a late genomic event responsible for tumor progression.

## Conclusions

Our findings provide a novel and mechanistic insight into circHNRNPU_603aa which is secreted into the BM microenvironment and promotes MM progression through regulating SKP2 exon skipping and subsequently competitively inhibiting c-Myc ubiquitin. CircHNRNPU_603aa may serve as a promising diagnostic marker and potential therapeutic target in MM.

## Supplementary Information

**Additional file 1.**

**Additional file 2.**

**Additional file 3.**

## Data Availability

All data in our study are available upon reasonable request. The RIP-seq data and Agilent SBC-ceRNA microarray chips data were deposited in GEO (GSE 174501 and GSE 174510).

## Abbreviations

APEX: Assessment of Proteasome Inhibition for Extending Remission
BM: Bone marrow
GEO: Gene expression omnibus
GEP: Gene expression profiling
IF: Immunofluorescence
IRES: Internal ribosome entry site
MS: Mass spectrometry
MM: Multiple myeloma
MGUS: Monoclonal gammopathy of undetermined significance
ORF: Open Reading Frame
OS: Overall survival
PFS: Progression-free survival
RIP-seq: RNA Immunoprecipitation sequencing
TT2: Total therapy 2
UPS: Ubiquitin-proteasome system
WGCNA: Weighted gene correlation network analysis
