# Supplementary material for: A novel protein encoded by circHNRNPU promotes multiple myeloma progression by regulating the bone marrow microenvironment and alternative splicing
Source: J Exp Clin Cancer Res. 2022 Mar 8;41:85. doi: 10.1186/s13046-022-02276-7 (PMC8903708; doi:10.1186/s13046-022-02276-7)
Supplement: Supplementary file 2 — Additional file 2. [file 13046_2022_2276_MOESM2_ESM.pdf]

**The detailed information of patient cohorts**

The detailed information of patient cohorts was as follows: samples in TT2, GSE2658, were pre-treatment of bone marrow aspirates from multiple myeloma patients; samples in GSE136337 were 436 newly diagnosed MM patients with the purpose of identifying a high-risk cellular signature in the multiple myeloma bone marrow microenvironment; samples in HOVON65, GSE19784, were bone marrow plasma cell samples from 320 newly diagnosed multiple myeloma patients included in a large multicenter, prospective, randomized phase III trial for the purpose of identifying relevant, molecularly defined subgroups.

APEX cohort (GSE9782) included samples from the patients with relapsed myeloma enrolled in phase 2 and phase 3 clinical trials of bortezomib for the purpose of assessing the feasibility of prospective pharmacogenomics research in multicenter international clinical trials of bortezomib. TT2 cohort (GSE31161) contained 88 relapsed cases from TT2 cohort by long-term following up of these patients.
