# Supplementary material for: A novel protein encoded by circHNRNPU promotes multiple myeloma progression by regulating the bone marrow microenvironment and alternative splicing
Source: J Exp Clin Cancer Res. 2022 Mar 8;41:85. doi: 10.1186/s13046-022-02276-7 (PMC8903708; doi:10.1186/s13046-022-02276-7)
Supplement: Supplementary file 3 — Additional file 3. [file 13046_2022_2276_MOESM3_ESM.pdf]

Supplementary images of colony formation

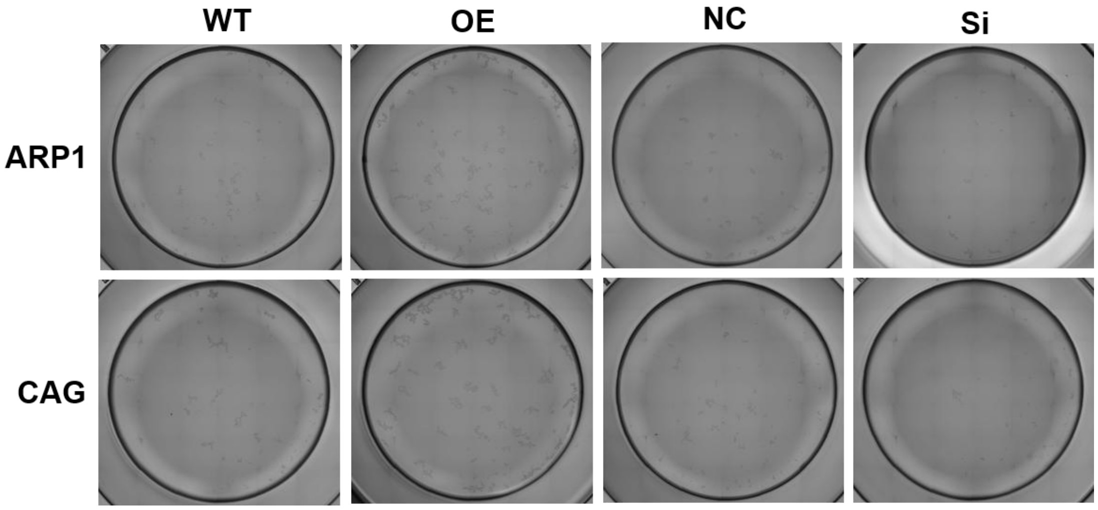

Figure S1 CircHNRNPU_603aa promotes MM cell clonal expansion. The whole well of soft agar plates showed more cell colonies formed by circHNRNPU-OE cells and less cell colonies formed by si-circHNRNPU cells compared with control cells.
